# The +Gz recovery of consciousness curve

**DOI:** 10.1186/2046-7648-3-9

**Published:** 2014-05-02

**Authors:** Typ Whinnery, Estrella M Forster, Paul B Rogers

**Affiliations:** 1 Oklahoma City, OK 73142, USA; 2 Mustang, OK 73064, USA

**Keywords:** Neurophysiology, Ischemia, Syncope, Acceleration, Consciousness, Unconsciousness

## Abstract

**Background:**

The limiting physiological envelope to extreme gravitational stress is defined by neurologic symptoms and signs that result from exceeding neurologic tolerance. The edge of the limiting envelope is defined by the complete incapacitation associated with acceleration (+Gz) induced loss of consciousness. Should + Gz-induced loss of consciousness occur in-flight, brisk recovery of conscious function is essential for aircraft recovery. If recovery does not occur, accident investigation aimed at preventing such accidents is enhanced by understanding the temporal aspects of the resulting incapacitation. The mechanistic basis of neurological reintegration leading to consciousness recovery is of broad medical and scientific interest.

**Methods:**

Recovery of consciousness episodes from a prospectively developed +Gz-induced loss of consciousness repository of healthy individuals was analyzed to define variables influencing recovery of consciousness. The time from loss to recovery of consciousness as measured by observable signs, is defined as the absolute incapacitation period. The absolute incapacitation period from 760 episodes of loss and recovery of consciousness in healthy humans was analyzed to define +Gz-profile variables that determine the duration of functional neurologic compromise.

**Results:**

Mean time from loss to return of consciousness for 760 episodes of consciousness recovery was 10.4 ± 5.1 s; minimum 1 s; maximum 38 s. Offset rate for the +Gz-exposure deceleration profiles varied from a minimum of 0.17 Gs^−1^ to a maximum of 7.93 Gs^−1^.The curve produced by plotting +G_z_-offset rate (Gs^−1^; *y*) versus absolute incapacitation period (s; *x*) described a hyperbolic relationship. The hyperbolic relationship indicates there is a minimum time (mean 8.29 ± 3.84 s) required for recovery of consciousness when complete loss of consciousness occurs.

**Conclusions:**

Mean recovery time from +Gz-induced unconsciousness is dependent on the deceleration profile's offset rate from the point of loss of consciousness. This relationship is described by a curve plotting offset rate and time for recovery of consciousness. This curve predicts when conscious function should return following exposure to +Gz stress sufficient to cause unconsciousness. The maximum +Gz level of the recovery exposure profile was found to be inadequate for predicting variations in the time for recovery of consciousness.

## Background

Environmental stress-induced incapacitation and unconsciousness limit human endeavors into extreme environments. Unconsciousness induced by exposure to acceleration (+Gz) stress envelopes in excess of the Earth's gravitational environment results from exceeding cardiovascular system capability to support nervous system function responsible for maintaining consciousness. When cardiovascular system support is exceeded, neurologic symptoms and signs, including unconsciousness, occur because of inadequate oxygenated blood flow above heart level. Quantitative description of the acceleration variables delineating the induction envelope of 888 unconsciousness episodes was previously provided [1]. Prevention of adverse outcomes from exposure to extreme acceleration environments also requires understanding +Gz recovery profiles, including the deceleration (offset) rate promoting optimum recovery of consciousness (ROC). The current study was conducted to define the time required for ROC along with acceleration variables determining ROC duration in healthy humans.

Determined efforts to prevent acceleration (+Gz)-induced loss of consciousness (G-LOC) are essential. In reality, however, complete prevention of G-LOC is doubtful [2-4]. Minimizing the duration of unconsciousness is part of optimal recovery from G-LOC and therefore a crucial aspect of operational safety. The results of the current study present the recovery envelope defining the induction of consciousness following 760 episodes of G-LOC. A primary goal was establishing the relationship between +Gz stress and ROC, including a recovery of consciousness (G-ROC) curve supplementary to the G-LOC curves [1].

Descriptions of the sign and symptom complex associated with G-LOC and G-ROC were developed early in acceleration research [5,6]. Previous studies defined the G-LOC syndrome to include the complete sign and symptom complex associated with loss and recovery of consciousness [7]. In acceleration research, the period of observable unconsciousness has been determined by conducting experiments and observing the time of the loss and recovery of consciousness based on observable symptoms and signs and experimentally defined as the absolute incapacitation period (ABSINCAP) rather than the unconsciousness period [8,9]. This formalized the established practice of describing unconsciousness occurring from the beginning of aviation medicine. Unconsciousness is an elusive concept. Utilization of the term absolute incapacitation was selected because loss and recovery of consciousness could be quantified by observable signs associated with unconsciousness and the observation that degradation of conscious function may occur prior to LOC and subsequent to ROC. The ABSINCAP time produced by specific acceleration (+Gz) profiles in various studies has been reported [10-15] and found to be a function of compromised cephalic nervous system (CPNS) blood flow duration [14,15]. Processes that shorten the ischemic period reduce ABSINCAP with longer periods of ischemia prolonging ABSINCAP. A specific correlation of ABSINCAP with the +Gz level of exposure when experimentally measuring +Gz tolerance, however, has not been found [5,16].

Quantitating G-ROC is a fundamental aspect of the education and training of individuals entering extreme environments where risk for G-LOC exists [17]. If protection fails to prevent G-LOC development of technology and methods to minimize G-ROC, including ABSINCAP, becomes essential [18-21]. Limiting the duration of unconsciousness requires comparison standards to evaluate successful ABSINCAP reduction [11]. The current findings should also assist medical and biomedical experts endeavoring to understand the characteristics of unconsciousness in normal humans, develop procedures to enhance rapid recovery of conscious function should unconsciousness occur, and define the safe envelope for exposure to environments that may induce unconsciousness.

## Methods

The subjects and experimental procedures for G-ROC were analogous to those previously described in the development of G-LOC curves [1].

### Subjects

G-LOC and G-ROC data from a centrifuge data repository prospectively developed to thoroughly describe the neurologic tolerance response of completely healthy humans to acceleration stress on human centrifuges was utilized [8,9,22]. The data from the centrifuge repository contained 888 G-LOC and 760 G-ROC episodes spanning 1978–1992 generated from centrifuge exposures at the USAF School of Aerospace Medicine; Brooks AFB, Texas and the Naval Air Warfare Center; Warminster, Pennsylvania. All experimental human research exposure to acceleration was approved by the advisory committees for human research at the respective institutions where the research was conducted: Naval Air Warfare Center (Advisory Committee for Human Experimentation) and USAF School of Aerospace Medicine (Committee for the Protection of Human Subjects). Data obtained from required military training were not attributable and did not require experimental consent. Subsequent database analyses were exempt in accordance with 45 CFR 46.101.

### Procedures

Although there were 888 G-LOC episodes, for this study, the data included all ROC episodes (760) that occurred *and* were completely described by both loss and recovery of consciousness kinetic parameters. If inadequate description of either G-LOC or G-ROC existed the episode was eliminated. The data therefore represented all completely described +Gz exposures of healthy subjects who experienced G-LOC and subsequent G-ROC during various types of +Gz profiles as previously described [1]. The subjects were in operational type aircraft ejection seats (back angle 13° to 30° tilt backward from vertical) and firmly secured upright with lap and shoulder restraints. This successfully held the individual's torso in the upright seat position in all but rare instances. Generally, there was some altered positioning of the head upon loss of postural muscle control, most frequently with the head dropping forward and laterally based on centrifuge movement from deceleration.

All runs were terminated immediately upon recognition of LOC. LOC was identified (defined) by sudden muscle relaxation (facial, extremities, and torso), abrupt change in facial expression (eye fixation, staring, blank expression), loss of postural tone, loss of response, and/or loss of required task performance. ROC was identified (defined) by recovery of muscle tone (facial, extremities, and torso), return of postural tone, response, and/or abrupt change in facial expression (purposeful eye movement, return of expression). These are the neurological signs that in combination signal loss of sensory input, motor output, and integrated central nervous system function defining complete LOC. They include the continuum of neurological compromise signs represented by compromise of motor and cognitive function from acceleration stress classified as being almost losing consciousness [23].

All G-LOC and G-ROC episodes, as recorded on videotape, were analyzed independently by at least two investigators with agreement on all measurements of G-LOC and G-ROC onset to within 1 s. Based on the technology available over the period of time that data collection transpired, high-resolution video recording of most LOC episodes was made with a minimum of two cameras, one focused specifically on the head and face and the second covering the entire body. The independent review was conducted at real time and slow motion to obtain measurements by each investigator. The ROC time, defined as ABSINCAP, was determined as the time from onset of G-LOC to the time of ROC. The +Gz level at the onset of G-LOC defined the maximum +Gz level for the recovery exposure profile (GMAX). The offset rate (G/s) was calculated using the time of offset from GMAX to the onset of base +Gz level. All offset rates were linear over the course of deceleration from GMAX except for the expected momentary transition points of change when leaving maximum +Gz and when attaining the final base +Gz (below +2Gz).

Immediate post recovery interviews, medical examination, and standardized loss of consciousness reports captured the experiences of the individuals both immediately and over the subsequent 24-h period of recovery for many of the exposures. The methods utilized are analogous to those utilized clinically [24] and aeromedically [12] as previously described for defining LOC episodes. They also remain the mainstay of determining states of consciousness even with the most sophisticated neurological monitoring technology in ideal clinical monitoring environments [25].

The acceleration exposures that resulted in unconsciousness were prospectively collected from years of on-going training and experimental research having a variety of specific exposure characteristics. This was required because LOC has not been an acceptable routine tolerance endpoint for experimentation on healthy humans. This avenue was the only method available to amass the number and exposure ranges to attempt adequate description of G-LOC and G-ROC. The +Gz levels, profiles, and offset rates were therefore those that were part of the standard operating centrifuge procedures for on-going training and research studies. There was no ability to have a pre-defined experimental design to evaluate the effect of various offset rates or other recovery parameters on G-ROC for the majority of the acceleration exposures. The study was therefore limited by the range of available offset rate and +Gz level data as well as the training and experimental +Gz-exposure profiles employed.

### Statistical analysis

The descriptive statistics reported include mean, minimum, maximum, standard deviation (SD), standard error (SE), range, median, and mode. Comparison analysis was conducted using analysis of variance (ANOVA) and *t* test statistics. *Post hoc* analysis was conducted using Tukey's honest significant difference (HSD). Statistical significance was established at alpha = 0.05.

A Shapiro-Wilk test was subsequently conducted on each of the data groups, as defined in Table 1, to test for normality. The Null hypothesis of this test was that the data is normally distributed with the alternate being the data is not normally distributed. A test of the data in each of the groups resulted in rejection of the null hypothesis indicating that the assumption of a normal distribution could not be accepted. A distribution-free Tukey multiple comparison test was therefore performed in such cases for data group comparisons. This non-parametric test relies on ranking the data and making a comparison of the distribution of the ranks between each group. Using SAS version 9.3, this was accomplished utilizing methods available in PROC RANK and PROC GLM [26]. This method is equivalent to the Kruskal-Wallis test which can be performed in PROC NPAR1WAY except it has the added advantage of being capable of performing multiple comparisons unavailable in the NPAR1WAY procedure. The level of significance was adjusted based on the number of comparisons made.

**Table 1:** Characteristics of absolute incapacitation period (ABSINCAP) for narrowly separated offset rate ranges

Offset rate interval (G/s)*	**Absolute incapacitation period (s)**							
	***N***	**Mean**	**SD**	**Min**	**Max**	**Range**	**Median**	**SE**
(0.1–0.499) (0.42 ± 0.07) (0.171–0.497)	136	13.61	5.26	3	29	26	13	0.45
(0.5–0.599) (0.54 ± 0.03) (0.50–0.599)	100	14.13	6.00	4	38	34	13	0.60
(0.6–0.699) (0.65 ± 0.03) (0.6–0.699)	43	9.47	3.83	2	21	19	9	0.58
(0.7–0.799) (0.74 ± 0.03) (0.7–0.797)	38	9.89	3.34	2	17	15	10	0.54
(0.8–0.899) (0.85 ± 0.04) (0.80–0.889)	64	9.34	3.84	2	24	22	9	0.48
(0.9–0.999) (0.95 ± 0.03) (0.90–0.983)	16	9.06	3.84	2	17	15	10	0.96
(1.0–1.099) (1.02 ± 0.03) (1.00)	50	9.44	4.97	2	27	25	10	0.70
(1.1–1.199) (1.14 ± 0.02) (1.1–1.18)	50	8.48	3.51	2	15	13	8	0.50
(1.2–1..299) (1.23 ± 0.03) (1.2–1.28)	35	8.63	4.35	2	19	17	8	0.73
(1.3–1.399) (1.34 ± 0.02) (1.3–1.38)	55	8.02	3.47	1	16	15	8	0.47
(1.4–1.499) (1.44 ± 0.04) (1.4–1.48)	13	8.54	3.55	3	15	12	8	0.98
(1.5–1.599) (1.55 ± 0.04) (1.5–1.59)	31	8.13	4.15	2	24	22	8	0.74
(1.6–1.699) (1.62 ± 0.03) (1.6–1.675)	19	7.47	3.11	2	14	12	7	0.71
(1.7–1.999) (1.85 ± 0.11) (1.7–1.975)	26	7.12	3.04	1	14	13	6.5	0.60
(2.0–2.99) (2.44 ± 0.30) (2.0–2.95)	27	7.96	3.38	2	16	14	8	0.65
(3.00–8.00) (3.95 ± 0.26) (3.6–7.93)	12	7.91	2.43	5	11	6	7	0.73
(0.1–8.00) (1.01 ± 0.68) All (0.17–7.93)	715	10.39	5.09	2.1	19.38	17.37	8.10	0.19

*For offset rate interval, the initial range represents the offset interval band, the middle number represents mean ± SD for the actual data interval band, and the last range the actual data range values within the offset interval band. Means for all offset rate intervals ≥0.6 G/s are not significantly different from each other and when combined have a mean for all levels ≥0.6 G/s of 8.53 ± 0.82 s.

### Curve analysis

Curve-fitting methods for nonlinear functions were utilized to develop the hyperbolic curve for the G-ROC data. The nonlinear model data analysis estimates parameters by the nonlinear least squares method. The parameters for the nonlinear model are identified and estimated using an iterative process commencing from the starting values provided for the model. An iterative process is required because there is no closed-form solution for parameter estimates. The iterative process converges to a solution minimizing the sum of squares from the data. This iterative process is a first-order Taylor series known as the Gauss-Newton method. The general form of acceleration strength-duration response data was shown to have the form of a rectangular hyperbola as follows:

$$y=\frac{m}{\left(x‒x_{\mathrm{ASYMPTOTE}}\right)}\;+y_{\mathrm{ASYMPTOTE}}$$

The asymptotes must be defined while the parameter ‘m’ is estimated from the data through the Gauss-Newton method. The shape of a hyperbola is defined entirely by its eccentricity (*ϵ*) with a rectangular hyperbola always having an eccentricity $\epsilon =\sqrt{2}$. The specific software used in nonlinear parameter estimation was the NLIN procedure implemented in SAS version 9.3. [26].

## Results

The duration of unconsciousness (ABSINCAP) resulting from G-LOC in the large (*N* = 760) healthy human population was defined. Analysis was then conducted separating the data into an offset rate dataset and a GMAX dataset. Return of blood flow to the CPNS depends on deceleration (offset rate) after consciousness is lost. Plotting offset rate versus ABSINCAP the relationship found defined a hyperbolic G-ROC curve indicating that ABSINCAP was related to the offset rate which then determines how rapidly CPNS blood flow is restored. A higher GMAX did have a trend to be associated with longer ABSINCAP; however, a curve relating GMAX with the ABSINCAP could not be developed.

### ABSINCAP description of the complete G-ROC dataset

**Figure 1:**
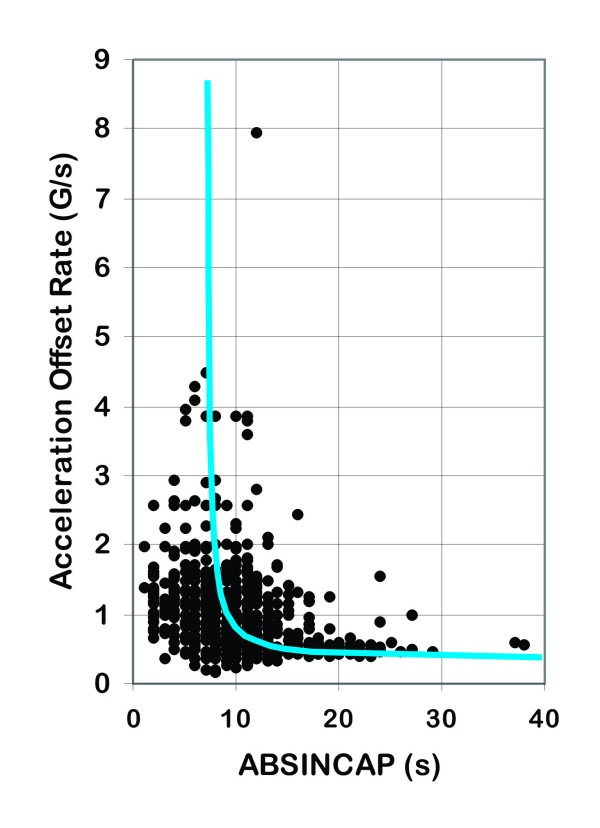
**Acceleration offset rate versus absolute incapacitation period from 715 recovery of consciousness episodes.** With overlay of hyperbolic +Gz recovery of consciousness curve (in blue) representing the mean absolute incapacitation period (ABSINCAP) for the experimental population.

**Figure 2:**
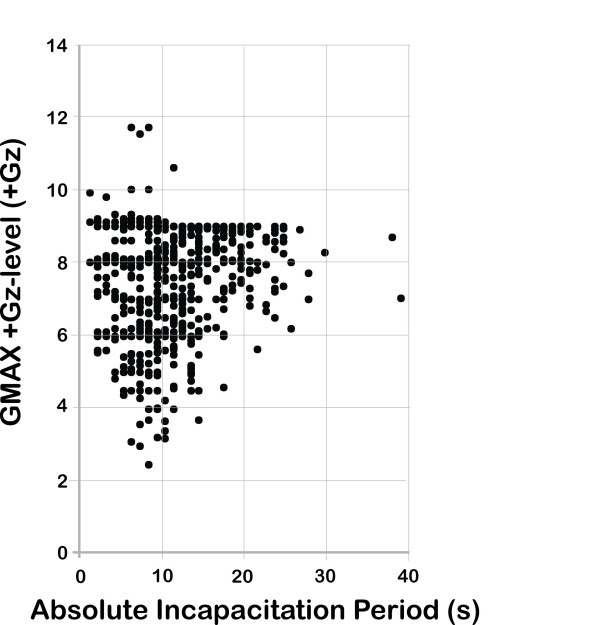
Maximum +Gz-level (GMAX) versus absolute incapacitation period from 760 recovery of consciousness episodes.

The entire population dataset consisted of 760 G-ROC episodes having an ABSINCAP of 10.4 ± 5.1 s (range 1–38 s). This ABSINCAP resulted from exposure to maximum +Gz levels from +2.5–+11.7 Gz and offset rates of 0.171–7.93 G/s. The offset rate dataset (*N* = 715 ROC episodes) had a mean offset rate of 1.01 ± 0.68 G/s resulting in a mean ABSINCAP of 10.40 ± 5.09 s (range 1–38 s) as shown in Figure 1. The GMAX dataset (*N* = 760 ROC episodes) had a mean maximum +Gz level of +7.70 ± 1.41 Gz (range +2.5–+11.0 Gz) resulting in a mean ABSINCAP of 10.36 ± 5.08 s (range 1–38 s) as shown in Figure 2.

### ABSINCAP as a function of offset rate

ABSINCAP was plotted as a function of offset rate for 715 G-ROC episodes as shown in Figure 1. To evaluate the effect of offset rate on ABSINCAP the offset rate dataset was parsed into narrow offset rate ranges with the resulting mean ABSINCAP determined for each offset rate range interval as shown in Table 2. For the most gradual offset rate ranges of 0.1 to 0.499 and 0.5 to 0.599 G/s, the ABSINCAP was 13.61 ± 5.26 and 14.13 ± 6.0 s, respectively. For the most rapid offset rate ranges of 2.0–2.99 and 3.0–8.0 G/s, the ABSINCAP was 7.96 ± 3.38 and 7.91 ± 2.43 s, respectively. ABSINCAP was therefore found to decrease as offset rate increases. The ABSINCAP means for these narrow ranges of acceleration offset rate of Table 2 indicated that a stimulus strength-duration relationship existed [27].

**Table 2:** Consolidation into groups (a–d) was accomplished on statistical basis of sample sizes necessary for multiple comparisons

**Group**	**Offset rate**	**Absolute incapacitation period (s)**						
	**interval (G/s)**^**o**^	***N***	**Mean**	**SD**	**Min**	**Max**	**Range**	**Median**
a*	(0.10–0.499) (0.42 ± 0.07) 0.17–0.497	136	13.62	5.26	3	29	26	13
b*	(0.5–0.999) (0.69 ± 0.14) 0.50–0.982	261	11.26	5.22	2	38	36	10
c**	(1.0–1.499) (1.20 ± 0.14) 1.00–1.48	203	8.60	4.05	1	27	26	8
d**	(1.50–7.93) (2.13 ± 0.90) 1.50–7.93	115	7.70	3.39	1	38	37	7
All	(0.10–7.93) (1.01 ± 0.68) 0.17–7.93	715	10.40	5.09	1	38	37	10

^**o**^For offset rate interval, the initial range represents the offset interval band, the middle number represents mean ± SD for the actual data interval band, and the last range the actual data range values within the offset interval band; *group ABSINCAP was significantly different from all other groups: *p* < 0.0001; **group ABSINCAP was significantly different from groups **a** and **b** but not significantly different from **c** or **d** respectively: *p* < 0.0001.

Consolidation of the data into four broad offset rate range groups was accomplished on the statistical basis of sample sizes necessary for multiple comparisons as previously described. The results of this separation are shown in Table 2. Analysis of these groups (**a** through **d**) revealed that the ABSINCAP for group **a** was different (*p* < 0.0001) from all the other groups. Group **b** was also different (*p* < 0.0001) from all the other groups. Groups **c** and **d** were not different (*p* < 0.0001) from each other; however, they were different (*p* < 0.0001) from groups **a** and **b**. This analysis illustrated that three offset ranges existed. Group **a** was therefore considered to represent a gradual offset rate (GOFF) range. Group **b** was considered to represent a transitional offset rate (TOFF) range, with groups **c** and **d** representing the rapid offset rate (ROFF) range of acceleration offset. Since the ABSINCAP for groups **c** and **d** was not different (*p* > 0.0001), ABSINCAP became independent of offset rate for rates ≥1.0 G/s, producing the minimum duration of unconsciousness (ABSINCAP) with a mean of 8.29 ± 3.84 s. When the offset rate was reduced below 1 G/s the ABSINCAP progressively increased (*p* < 0.0001) over the range between <1.0 and 0.5 G/s. Onset rates below 0.5 G/s were considered to be gradual with ABSINCAP continuously increasing as onset rate decreased. The above analysis supported a hyperbolic strength-duration relationship existing between offset rate and ABSINCAP. The G-ROC curve with ABSINCAP as a function of offset rate was developed using the least squares methods as previously described. The curve was derived by the best fit using the entire dataset shown in Figure 1. The resulting equation describing the hyperbolic relationship was

$$y=\frac{1.05}{\left(\mathrm{ABSINCAP}‒7.04\right)}+0.42$$

The pseudo coefficient of determination was *R*^2^ = 0.3461 with a standard error of the estimate for ‘*m*’ of 0.692 and a 95% confidence interval of 0.9133 and 1.1852 where ‘*m*’ is the parameter estimated in the Gauss-Newton-based algorithm of nonlinear regression analysis directly that is related to the eccentricity of the rectangular hyperbola. The resulting population ABSINCAP versus offset rate G-ROC curve was overlaid on the complete dataset presented in Figure 1.

### ABSINCAP as a function of +Gz exposure level

The G-ROC data points were also plotted with ABSINCAP as a function of GMAX as shown in Figure 2. No obvious hyperbolic relationship between these variables was observed. However, as the +Gz level increased, the distribution of ROC data points did spread toward longer ABSINCAP values. To evaluate the effect of GMAX on ABSINCAP, the data was separated into different +Gz-level ranges as shown in Table 3. Nonparametric analysis revealed that the ABSINCAP at +5 to <+6 Gz was significantly different (shorter) than the ABSINCAP at +7 to <+8 Gz (*p* < 0.01) and +8 to ≤9 Gz (*p* < 0.05). The ABSINCAP therefore did show a significant lengthening as GMAX increased; however, it was not a continuous trend for all +Gz-level ranges. There was inadequate data to evaluate the ABSINCAP for +Gz levels >+9.0 Gz. The maximum value of ABSINCAP for the integer ranges of GMAX was also consistent with an increased ABSINCAP trend up to +9 Gz as observed in Table 3. The evaluation of ABSINCAP as a function of GMAX did not allow curve development and based on the available dataset can only be characterized by the overall mean value for the ABSINCAP being 10.36 ± 5.08 s (range 1–38 s).

**Table 3:** Characteristics of absolute incapacitation times for maximum +Gz level of recovery exposure profile (GMAX) separated into +Gz-level ranges

+Gz level*	**Absolute incapacitation period (s)**							
	***N***	**Mean**	**SD**	**Min**	**Max**	**Range**	**Median**	**SE**
(2.5–4.99) (4.14 ± 0.64) (2.5–4.96)	33	9.18	2.94	4	17	13	9	0.51
(5.0–5.99) (5.39 ± 0.30) (5.0–5.93)	48	8.40	3.78	2	21	19	8	0.55
(6.0–6.99) (6.28 ± 0.30) (6.0–6.99)	104	9.98	4.37	2	25	23	10	0.43
(7.0–7.99) (7.29 ± 0.34) (7.0–7.99)	149	11.25	5.89	2	38	36	10	0.48
(8.0–8.99) (8.29 ± 0.32) (8.0–8.96)	196	11.76	5.89	1	37	36	11	0.42
(9.0–12.0) (9.34 ± 4.05) (9.0–9.99)	230	9.08	4.09	1	24	23	9	0.27
(2.5–12.0) (7.69 ± 1.41) All (2.5–11.7)	760	10.36	5.08	1	38	37	10	0.18

*For the +Gz-level interval, the initial range represents the +Gz-level interval band, the middle number represents mean ± SD for the actual data interval band, and the last range the actual data range values within the +Gz-level interval band.

## Discussion

Loss and recovery of consciousness kinetics confirm, as would have been predicted, that compromise of neurologic function is related to the ischemic insult characteristics associated with both acceleration and deceleration.

### ABSINCAP as a function of offset rate

The relationship between offset rate and the ABSINCAP suggests that return of blood flow to the CPNS resulting from a rapid offset of +Gz stress reduced the ABSINCAP and a slower return of blood flow resulting from slower offset of +Gz stress produced a longer ABSINCAP. Rapid offset (ROFF), representing rates ≥1.0 G/s, produced shorter ABSINCAP (8.29 ± 3.84 s); transitional offset (TOFF), represented by rates <1.0 and ≥0.5 G/s, produced gradually changing (increasing) ABSINCAP (11.26 ± 5.22 s); and gradual offset (GOFF), represented by rates <0.5 G/s, produced longer ABSINCAP (13.62 ± 5.26 s). The G-ROC data provided in Table 1 and the curve developed as shown in Figure 1 reveal that for ROFF, ABSINCAP becomes independent of offset rate with offset rates ≥1.0 G/s. ABSINCAP gradually begins to increase (mean 9.59 s) with TOFF between 1.0 and 0.5 G/s. With rates less than 0.5 G/s (GOFF) ABSINCAP increases continuously as the offset rate slows. GOFF recovery is prolonged with a mean of 13.83 s for all G-ROC episodes with offset rates <0.5 G/s. The longest ABSINCAP values of 29 and 38 s occurred for the slowest offset ranges as shown in Table 1. With continuously prolonged ischemia of the CPNS resulting from increasingly slower offset rates, prolonged functional recovery would be predicted to eventually merge with insults that compromise CPNS integrity and ultimately death.

The G-ROC single-line curve represents the complete study population's (760 G-ROC episodes) least squares estimation of ABSINCAP (10.4 ± 5.1 s). The actual data points reflect the distribution of responses from the available population. The distribution of the data points range from minimum through maximum values (1 to 38 s) for ABSINCAP over the range of offset rates and +Gz levels was defined in Tables 1, 2, and 3. These distributions may be an indication of individual human variability to +Gz-induced ischemia within the population or they may reflect other factors, including the human ability to make accurate measurements of the loss and recovery of consciousness. Experimental studies focused on losing consciousness have only one endpoint (LOC) that necessitates determination of a change in conscious state. Experiments that deal with ROC have two endpoints (both LOC and ROC) that necessitate determination of changes in the state of consciousness. Human recognition limitations in measuring states of consciousness should be recognized in assessing the results of all LOC and ROC experiments.

### ABSINCAP as a function of +Gz level

The relationship between the +Gz level of exposure when G-LOC occurred revealed a more complex relationship with the ABSINCAP associated with G-ROC. The mean ABSINCAP did have small but significant increases in duration as the +Gz level increased as shown in Table 3. It is evident in Figure 2 that the ABSINCAP range widens as the +Gz level increased indicating that as the +Gz level increased, some individuals were exposed to +Gz profiles that increased the ischemic insult to the CPNS and therefore resulted in a longer time (ABSINCAP) for recovery.

When the majority of the individuals in a study population are exposed to +Gz levels just above their tolerance level, this equates to the individuals being taken to a similar level of CPNS ischemia based on their individual tolerances even though the magnitude of the exposure levels (the +Gz level) may be considerably different. With equivalent ischemic insults induced, the ABSINCAP would also be predicted to be equivalent.

More than a single underlying process may be operative during recovery. To attain high +Gz levels requires anti-G protection including the performance of an anti-G straining maneuver (all out muscular tensing and respiratory maneuvers to increase intrathoracic pressure) and wearing some type of anti-G suit/protective ensemble. This allows attainment of higher levels of +Gz compared to relaxed, unprotected conditions. In the protected condition a high +Gz level may be attained during G-LOC induction; however, when LOC occurs, the complete loss of motor control suddenly leaves the individual unprotected far above relaxed tolerance. The anti-G suit as well is deflated based on a specific deflation profile proportional to the G-offset rate [11]. Complete loss of all the protection that allowed attainment of very high +Gz, immediately on LOC, leaves the individual well above unprotected, with relaxed tolerance, and may be associated with an increased ischemic insult and prolonged ABSINCAP.

### Comparison with previous studies

No previous G-ROC curves have been developed. Absence of and/or variability in defining LOC and ROC kinetic measurements make strict comparisons complex between studies. The majority of studies provide only a single value for all ROC episodes in the study population. Human studies, for safety reasons, expose individuals close to their tolerance levels and also eliminate the ischemic stress immediately upon exceeding the ischemic tolerance producing LOC. This is also true for most instances of naturally (terrestrial) occurring LOC episodes; when neurologic tolerance is exceeded, loss of postural tone rapidly results in eliminating the +Gz stress (falling to horizontal). This has the unifying effect of making such ischemic insults of near-equal magnitude and therefore predicted to produce similar incapacitation periods. Determination of the resulting duration of unconsciousness was measured utilizing ABSINCAP and thus did not include the additional time for full reorientational recovery (period of confusion and disorientation; relative incapacitation) [9,12,14].

Rossen, Kabat, and Anderson (utilizing a cervical pressure cuff to induce immediate onset strangulation in physiological healthy subjects) found no significant correlation between time of recovery with time of eye fixation or with duration of anoxia [28]. For their 28 LOC episodes defining their ABSINCAP equivalent, the mean was 6.14 s [29]. The measurements of ROC time included the time required to respond to light-buzzer warning signals which would be analogous to what previous G-ROC kinetic analyses would classify as the total incapacitation period [9,12,14]. They considered the variance in recovery times to be related chiefly to psychological factors.

The excellent Canadian G-LOC study of Kerr and Russell did not report analysis of ROC as a function of offset rate; however, they did report finding no temporal relationship between the +Gz level of exposure and ROC duration. Based on the +Gz profiles inducing LOC being part of incremental +Gz increases to determine tolerance, they were not surprised by the absence of an increased incapacitation associated with increasing +Gz level [5]. Recovery of consciousness was reported as the number of ROC responses within 5-s intervals. Means were plotted for each integer +Gz level (+3 to +10 Gz) from 360 centrifuge runs on 95 healthy subjects with offset rates varying from 0.4 to 1.8 G/s. The mean duration of unconsciousness was 12 s with a range of 3 to 60 s. Reanalysis of the Kerr-Russell data consisting of the duration of unconsciousness being reported at 5-s intervals, revealed a trend for G-ROC to increase from approximately 13 to 20 s as the +Gz level increased from +3 to +10 Gz. The lengthening ABSINCAP once again suggests that exposure to higher +Gz profiles increases the potential for increased ischemic insult and a longer time (ABSINCAP) for recovery.

The baboon (*N* = 7) study by Burns et al. consisting of 92 G-LOC episodes utilized electroencephalographic criteria and found the unconsciousness duration to be 8.2 ± 3.2 s with a range of 2.8 to 23.4 s [10]. Statistical analysis applied to Figure nineteen of the Stauffer acceleration study utilizing US Navy personnel (*N* = 215) over 196 G-LOC episodes found the unconsciousness time to be 14.39 s with a range of 1 to 38 s [30]. The Lempert et al. clinical characteristics study utilizing humans in 42 LOC episodes found the unconsciousness time to be 12.1 ± 4.4 s with a range of 4.5 to 21.7 s [24]. The values from the above studies should be compared with the value for the entire group (760 G-LOC episodes) found in the current study to be 10.4 ± 5.1 s.

### Overall neurophysiology of G-LOC and G-ROC

Comparing the findings of G-LOC and G-ROC, it is apparent that for both the induction of unconsciousness and consciousness, there is a required ‘time constant’ associated with both neurologic state transitions. This is reflected by both exhibiting a hyperbolic curve for the onset and offset rates associated with changing the neurologic state from consciousness to unconsciousness and unconsciousness to consciousness, respectively. For G-LOC, the functional buffer period was considered to be the period of time that the brain could maintain neurologic function when CPNS blood flow was immediately reduced below a critical support level [1,31,32]. The functional buffer period is a component of the loss of consciousness induction time (LOCINDTI). For G-ROC, there similarly appears to be a period of time, a recovery buffer period, which exists prior to integrated neurologic function being restored.

The limiting time for rapid onset of G-LOC was 10.40 s. The limiting time for rapid offset of G-ROC was 8.29 s. When the exposure characteristic differences of the induction and recovery events are taken into consideration the times become very similar. This close kinetic symmetry suggests that there may be similar mechanisms underlying the energetics transpiring when critical reduction of neurologic blood flow results in G-LOC and likewise the requirements when blood flow returns prior to G-ROC. Both appear to have buffer periods. It is clear that no pathologic changes have been documented to result from these types of transient ischemic exposures clinically [33-35]. The kinetic results of G-LOC and G-ROC represent a safely tolerated ischemic envelope.

For G-LOC, the functional buffer period serves to safely (without pathologic insult) maintain consciousness during the frequent, transient episodes of loss of adequate blood flow to the CPNS experienced daily in the terrestrial environment. Should unconsciousness occur, the G-ROC recovery buffer period serves to ensure that full integrated conscious function is recovered before locomotion is restored in the hazardous environment that induced unconsciousness. Both buffer periods might be viewed to serve as protective mechanisms to ensure the absence of pathologic damage as well as organismal survival in the gravitational environment. The combined effects of different +Gz onset and offset rate profiles used to induce unconsciousness have previously been shown to have an effect on the incapacitation duration; however, they were not evaluated in this study [9,11,14,15]. Integration of the results of loss and recovery of consciousness is a required step that should lead to a complete description of +Gz-induced unconsciousness.

### Applications

ABSINCAP defines normal recovery time should LOC occur and therefore represents a standard for accident timeline reconstruction when LOC is investigated as being contributory to accident or incident causation. Life or death in extreme environments may be differentiated by recovery times differing by only a few seconds. The G-ROC curve provides support for using offset rates ≥1.0 G/s in research studies to ensure the minimum ischemic insult and the shortest ABSINCAP are achieved. Understanding the normal ROC response kinetics to ischemic/anoxia in healthy humans may provide diagnostic clues to separate otherwise healthy individuals from those with clinical cardiovascular (dysrhythmias) or neurologic (epilepsy, altered autonomic responses) irregularities [36,37]. Clinical history taking is considered the cornerstone of diagnosis in patients presenting with transient LOC as well as distinguishing between various forms of syncope based on the kinetics of the unconsciousness event [38]. Wieling considered diagnosis begins with ‘the setting of the event, any provocations or triggers, clinical symptoms and signs of the prodromal phase, progressing through the period of actual unconsciousness, up to and including the recovery phase are all crucial elements.’ [39]. Understanding the kinetics of loss and recovery of consciousness provides insight into the mechanisms of consciousness, unconsciousness, and the transitions between these different neurologic states. This understanding includes how we have successfully evolved and now survive in a gravitational environment with a neurologic system located above a supporting cardiovascular system.

## Conclusions

In healthy humans, the mean time for G-ROC when G-LOC occurs is 10.40 s. When the offset rate following G-LOC is increased, G-ROC occurs more rapidly as defined by a hyperbolic relationship with offset rate defining a limiting mean ABSINCAP of 8.29 s when offset rates are ≥1.0 G/s. Higher +Gz levels increase the duration of unconsciousness; however, the time for G-ROC is dependent on factors other than simply the +Gz level of exposure. The results of studies associated with the response to the extreme acceleration environment have aeromedical applications relative to aircrew response to acceleration stress as well as medical applications where the temporal response to loss and recovery of consciousness is an important aspect of clinical diagnosis.

## Availablity of supporting data

None.

## Abbreviations

+Gz: the standard terminology used for describing acceleration stress on the human when the direction of the acceleration is headward and the direction of the resultant inertial force is footward; ABSINCAP: absolute incapacitation period (unconsciousness period); CPNS: cephalic nervous system; G-LOC: +Gz-induced loss of consciousness; G-ROC: +Gz recovery of consciousness; GMAX: maximum +Gz level of the recovery exposure profile; GOFF: gradual offset rate/run; LOC: loss of consciousness; LOCINDTI: loss of consciousness induction time; ROC: recovery of consciousness; ROFF: rapid offset rate/run; TOFF: transitional offset rate/run.

## Competing interests

All authors declare they have no competing interests.

## Authors' contributions

TW and EMF in collaboration designed the study, conducted the data development, refinement, and analysis followed by initial manuscript development. PBR in collaboration with EMF concentrated on the statistical analysis and curve fitting. TW, EMF, and PBR all contributed to the composition of the manuscript followed by reading and approving the final manuscript.
